# PKM2 promotes lymphatic metastasis of hypopharyngeal carcinoma via regulating epithelial-mesenchymal transition: an experimental research

**DOI:** 10.1186/s13000-024-01474-5

**Published:** 2024-03-02

**Authors:** Xin Zhou, Yanshi Li, Min Pan, Tao Lu, Chuan Liu, Zhihai Wang, Fengxiang Tang, Guohua Hu

**Affiliations:** 1 Department of Otolaryngology, The First Affiliated Hospital of Chongqing Medical University, 1 Youyi Road, Yuzhong District, Chongqing, 400016 China; 2 Department of Otolaryngology, Chongqing Traditional Chinese Medicine Hospital, Chongqing, China

**Keywords:** Hypopharyngeal carcinoma, Lymphatic metastasis, Pyruvate kinase M2, Epithelial-mesenchymal transition, Foot-pad xenograft model

## Abstract

**Background:**

Patients with hypopharyngeal carcinoma (HPC) have a poor prognosis mainly because of lymphatic metastasis. This research aimed to determine the PKM2 role in lymphatic metastasis in HPC and the underlying molecular mechanism contributing to this phenomenon.

**Methods:**

PKM2 in HPC was studied for its expression and its likelihood of overall survival using TCGA dataset. Western blotting, qRT-PCR, and IHC were employed to confirm PKM2 expression. Methods including gain- and loss-of-function were used to examine the PKM2 role in HPC metastasis in vitro and in vivo. In vitro and in vivo studies also confirmed lymphatic metastasis’s mechanism.

**Results:**

Prominent PKM2 overexpression was seen in patients with lymphatic metastasis of HPC, and there was an inherent relationship between a high PKM2 level and poor prognosis. In vitro research showed that knocking down PKM2 decreased tumor cell invasion, migration, and proliferation while promoting apoptosis and inhibiting epithelial-mesenchymal transition, but overexpressing PKM2 had the reverse effect. Animal studies suggested that PKM2 may facilitate tumor development and lymphatic metastasis.

**Conclusions:**

Our findings suggest that PKM2 may be a tumor’s promoter gene of lymphatic metastasis, which may promote lymphatic metastasis of HPC by regulating epithelial-mesenchymal transition. PKM2 may be a biomarker of metastatic potential, ultimately providing a basis for exploring new therapeutic targets.

## Introduction

According to the American Cancer Society, head and neck squamous cell carcinoma (HNSCC) is the world’s sixth most frequent malignant tumor in 2022, accounting for 5.3–7.1% of all systemic malignancies [1]. Because of its widespread distribution, it poses a major threat to public health across the world. Cigarette smoking, excessive alcohol use, and exposure to certain viruses, including Epstein-Barr virus and human papillomavirus, are the leading risk factors (RFs) for developing HNSCCs [2, 3]. The oral cavity, nasal cavity, sinuses, nasopharynx, oropharynx, hypopharynx, and larynx all have squamous mucosa affected by HNSCC. Malignant tumors of the hypopharynx are among the deadliest to develop in the head and neck area. Early clinical signs are not normal and are often misinterpreted because of the unique anatomical structure. Surgery, radiation therapy, and systemic chemotherapy are the mainstays of current care. Lymphatic metastasis (LM) was shown to be present in 65–80% of hypopharyngeal carcinoma (HPC) patients at the time of diagnosis, even though diagnostic tools and treatment protocols have greatly improved over the years [4, 5]. Patients with HPC often present with advanced disease.

The spread of malignant tumors, or metastasis, is crucial in determining the best course of therapy, the effectiveness of that treatment, and the survival of a patient’s life. LM is the most prevalent metastasis linked to lymphangiogenesis, tumor cell proliferation, invasion, migration, and the release of lymphangiogenic cytokines [6]. According to many studies, patients with lymph node metastases from various cancers have a poor prognosis and a lower overall survival (OS) [7, 8]. The presence of metastatic disease in the cervical lymph nodes is an independent RF for the HPC patients’ prognosis. The prognosis of HPC is affected by LM in terms of lymph node diameter, metastatic lymph node number, and extracapsular dissemination [9, 10]. Despite extensive research aimed at identifying biomarkers associated with LM in HNSCC [11, 12], little is known about the molecular pathways involved [13, 14], and patients with LM do not do well with therapy advances [15].

In order to study the mechanism of LM of HNSCC, our team, as a regional research center of head and neck surgery in southwest China, carried out a series of in-depth research work [16–19]. Our team has previously employed transcriptome sequencing to identify differentially expressed genes between patients with and without LM in HPC. One of these genes, pyruvate kinase M2 (PKM2), shows notable differential expression. These findings point to a possible function for PKM2 in cancer metastasis via the lymphatic system. One of the essential enzymes in the glycolytic process is pyruvate kinase, which converts phosphoenolpyruvate into pyruvate and adenosine triphosphate (ATP). There are four isoenzymes of PK; PKR, PKL, PKM1, and PKM2 [20]. PKM2 is well expressed in rapidly dividing cells, such as those seen in embryos and tumors. Since Christofk’s discovery that expression of PKM2 is required for cancer-specific aerobic glycolysis (Warburg effect), there has been much interest in the functions of PKM2-involved cancer formation [21]. PKM2 has a role in tumor metabolism, oncogenic cytokinesis, tumor metastasis, and tumor development [22]. PKM2 is a protein kinase that phosphorylates its substrates to control gene transcription [23], and it has been shown to have an essential role in cancer cell proliferation and survival in previous research [24, 25]. To identify therapeutic targets and create new therapeutics for primary and metastatic HPC, a better knowledge of the biochemical roles of PKM2 in tumor progression is essential.

Epithelial-mesenchymal transformation (EMT) has been proven as an essential process in the metastasis and invasion of various malignancies [26]. EMT promotes tumor metastasis by enhancing invasion, anti-apoptosis, and diffusion abilities [27]. Upregulation of mesenchymal markers, downregulation of epithelial protein markers, and loss of cell polarity and cell-cell adhesion are some of the hallmarks of EMT [28]. Several signaling pathways are involved in EMT, including PI3K/AKT/mTOR, transforming growth factor β (TGF-β), and Wnt. The complex interactions between cells, microenvironment and various signaling pathways facilitate the metastatic progression from the tumor in situ to invasive and aggressive carcinoma.

Upregulation of PKM2 in clinical samples of HPC was observed, which was shown to be associated with a poor prognosis. Based on these findings, we hypothesized that PKM2 regulated FaDu cell viability and migration. By examining the protein level of the EMT marker and EMT transcription factor (EMT-TF) mRNA expression, we further demonstrated that PKM2 mediates EMT to increase the invasion and migration of FaDu cells. Our study’s results may guide future efforts to identify biomarkers for PKM2 and develop therapeutic medicines aimed at this protein.

## Materials and methods

### Bioinformatics

From the TCGA data portal (https://portal.gdc.cancer.gov/), the RNA sequencing dataset of 500 HNSCC samples, including transcriptome data and complete clinical information, were downloaded. After all the data sets were standardized, the R software package “survival” and “survminer” were used to calculate the OS rate of HNSCC patients in the high and low PKM2 expression groups using the “Kaplan Meier” method and visualized [29].

### Patients and samples

HPC tumor tissues and surrounding normal tissues were taken from surgery patients at the First Affiliated Hospital of Chongqing Medical University between 2012 and 2019. The following criteria were set for participation: The cancer was identified as hypopharyngeal squamous cell carcinoma by two experienced pathologists; To gauge the severity of the lesions before surgery, ultrasonography, CT, or MRI scans were conducted; There was no evidence of distant metastases or hemorrhagic metastasis; No other forms of cancer were present at the time of the first diagnosis of hypopharyngeal carcinoma; no prior radiotherapy, chemotherapy, or targeted therapy were administered. Protein and RNA extraction required the fresh specimens to be frozen in liquid nitrogen and kept at -80 °C. Formalin was used to preserve other specimens, and those were subsequently embedded in paraffin. The specimen collection was conducted per the Helsinki Declaration, and the Institutional Ethics Committee of the First Hospital of Chongqing Medical University authorized the plan. Each patient signed a written consent form. Table 1 displays the patients’ clinical features.

**Table 1:** The clinicopathological features of patients with hypopharyngeal carcinoma

Features	Variables	No. (%)
Age	≥ 60	36 (60)
	< 60	23 (40)
Smoking	Yes	55 (93.2)
	No	4 (6.8)
Gender	Male	58 (98.3)
	Female	1 (1.7)
Pathological stage	Early (T1-T2)	13 (22)
	Advanced (T3-T4)	46 (78)
Lymphatic metastasis	Presence	38 (64.4)
	Absence	21 (35.6)
Pathological differentiation	Low	13 (22)
	Moderate and high	46 (78)

### Extraction of RNA and quantitative real-time PCR (qRTPCR)

Total RNA was extracted from HPC tissues and cells using the E.Z.N.A.® Total RNA Kit I (Omega, USA). PrimeScript RT kit (Takara, Dalian, China) was used for the reverse transcription (RT), and SYBR primescript RT-PCR kit (Takara) was used for the qRT-PCR. The PCR amplification settings were 40 cycles of 30 s at 95 °C, 5 s at 95 °C, and 1 min at 60 °C. Glyceraldehyde 3-phosphate dehydrogenase (GAPDH) was set as an internal control. 2^−ΔΔ^Ct was used to determine the relative expression. Table 2 displays the primer sequence.

**Table 2:** Primer sequences

Gene name	Primer sequences (5′-3′)
PKM2	Forward: TGACGAGAACATCCTGTGGC
	Reverse: TTTTCCACCTCCGTCACCAG
E-cadherin	Forward: TGCCCAGAAAATGAAAAAGG
	Reverse: GTGTATGTGGCAATGCGTTC
N-cadherin	Forward: GACAATGCCCCTCAAGTGTT
	Reverse: CCATTAAGCCGAGTGATGGT
Vimentin	Forward: GAGAACTTTGCCGTTGAAGC
	Reverse: GCTTCCTGTAGGTGGCAATC
Snail	Forward: GCGAGCTGCAGGACTCTAAT
	Reverse: CCTCATCTGACAGGGAGGTC
Slug	Forward: TGATGAAGAGGAAAGACTACAG
	Reverse: GCTCACATATTCCTTGTCACAG
GAPDH	Forward: CCTCTGACTTCAACAGCGAC
	Reverse: TCCTCTTGTGCTCTTGCTGG

### Protein extraction and western blotting

HPC tissue specimens and treatment cell lysis were performed using a total protein extraction kit (KeyGen BioTECH, Jiangsu, China), and western blotting analysis was performed per protocol. Specifically, a BCA assay kit (Beyotime, Shanghai, China) was used to quantify proteins. Following separation by 10% SDS-PAGE (Beyotime), protein lysates were transferred to a PVDF membrane for analysis (Beyotime). Overnight at 4℃, the following primary antibodies were used: anti-PKM2 (1:1000, #4053; CST,USA), anti-Slug (1:1000, #9585; CST), anti-Snail (1:1000, #3879; CST), anti-vimentin (1:1000, #5741; CST), anti-N-cadherin (1:1000, #13,116; CST), anti-E-cadherin (1:1000, #3195; CST), and anti-GAPDH (1:3000, ab-181,602; Abcam, UK). The anti-Rabbit goat IgG secondary antibody (1:5000, Beyotime) was incubated for 1 h at room temperature. The blotting was visualized using an Enhanced Chemiluminescence (ECL) kit (Termo, Shanghai, China), and images were obtained using a ChemiDoc Touch Imaging System (Bio-Rad, USA). As an internal control, GAPDH was used.

### Immunohistochemistry (IHC)

Fifty-nine cases of HPC were analyzed using IHC on paraffin-embedded (4 μm) tissue slices. After dewaxing in fresh xylene, paraffin slices were hydrated in gradient ethanol. Citric acid buffer at 100 °C for 30 min was used to repair the antigen. Each segment was incubated with a primary antibody for 12 h at 4 °C after being blocked with an endogenous peroxidase inhibitor for 15 min at 37 °C. The slices were rinsed three times in phosphate-buffered saline (PBS) after the second antibody reaction at 37 °C for 20 min and then stained with diamino diphenylamine (DAB; ZSGB-BIO, China) and counterstained with hematoxylin. Instead of using a glass slide treated with the primary antibody, PBS was utilized as the negative control. Two highly qualified pathologists independently evaluated each slide. PKM2 expression was classified as either high (moderate or strong staining, > 50% of tumor cells) or low (no staining or relatively mild staining, ≤ 50% of tumor cells) based on the intensity and area of staining.

### Cell culture

The FaDu cell line was obtained from the Center for Molecular and Cell Science, Chinese Academy of Sciences (Shanghai, China). FaDu cells were cultured in DMEM high-glucose media (Gibco, USA) supplemented with 10% fetal bovine serum (FBS) (Gibco, USA) and 1% penicillin-streptomycin (Beyotime) at 37 °C in a 5% CO_2_ humidified incubator.

### Lentivirus transfection

Lentivirus carrying green fluorescent protein (GFP) was purchased from GeneChem (Shanghai, China). FaDu cells were transfected with lentivirus short hairpin RNA (shRNA) and an overexpression vector to knock down and overexpress PKM2. On day 2, when cell growth was around 50–60%, they were injected in a 6-well plate at 1 × 10^6^ cells/well density and transfected with lentivirus. The multiplicity of infection (MOI) was 10. Eight hours after transfection, transfected cells were grown for 48 h. The cells were then cultured in a solution with 2 µg/mL purinomycin (Beyotime, Shanghai, China) to produce stably infected cells. Western blotting and qRT-PCR examined the expression of PKM2 on the collected cells.

### EdU proliferation assay

EdU assay kit (RiBoBio, Guangzhou, China) was used to quantify cell proliferation. Six-well plates were seeded with transfected FaDu cells (sh-PKM2, sh-NC, PKM2, vector). When cell growth had reached 80%, we followed the manufacturer’s instructions with one exception: we switched from Hoechst 33,342 to DAPI (Beyotime) to stain the nuclei. Rapid analysis of the staining was performed using an inverted fluorescent microscope. EdU positive expression rate = number of EdU positive cells/total cell number ×100%.

### Flow cytometry

FaDu cells that had been transfected were washed in PBS and then digested with trypsin. They were stained with annexin V-FITC/PI in a binding buffer as directed by the manufacturer. Flow cytometry (FCM) (Biosciences, CA, USA) was used on the samples to check for apoptosis. Trypsin was used to digest the transfected cells before they were fixed with 70% ice ethanol in a refrigerator at -4 °C overnight. After treating the cells with RNase A and propidium iodide, FCM was used to determine where in the cell cycle each sample was.

### Transwell migration and invasion assay

The 8 μm thick porous membrane in a 24-well plate (Corning, CA, USA) was used for the cell migration assay. FaDu cells (5 × 10^4^ cells/well) were introduced to the upper lumen after being suspended in the FBS-free DMEM medium and 15% FBS-containing DMEM medium in the bottom chamber. After 24 h, the cells were fixed in 4% polymethanol and stained with 0.5% crystal violet on the underside of the infiltrated membrane. Images were captured using an inverted fluorescent microscope, and invasive cell count was measured in five randomly selected fields. Matrigel (Biosciences, MA, USA) was used again in a Transwell assay to assess cell invasion.

### Wound healing assay

In 6-well plates, 1 × 10^6^ FaDu cells were seeded per well, and the cells were grown to near 100% confluence under standard conditions. 3–5 parallel scratches on the petri dish bottom were made using the tip of the 200 µL pipette. After that, the cells were maintained in serum-free media. The migration of cells was observed by microscope at 0 and 48 h after the scratch.

### Footpad tumorigenesis-inguinal popliteal lymph node metastasis (LNMs) model

An animal model of popliteal LNMs was developed at Chongqing Medical University using male BALB/c mice (Tengxin, Chongqing, China) at 5 weeks of age. Mice were randomized into two groups (10 mice in each). Subcutaneous injections of 1 × 10^6^ stably infected cells (sh-NC, sh-PKM2) in 0.1 ml PBS per animal were made into the foot pad. The main tumors and metastatic lymph nodes in the foot pad were collected when the mice were euthanized, and their volumes and weights were calculated. For HE and IHC staining, the tumors were paraffin-embedded. The tumor volume was calculated as: tumor volume =[length * (width)^2^] /2. The Ethics Committee of the First Affiliated Hospital of Chongqing Medical University approved all experiments on mice that were conducted per the Animal (Scientific Procedures) Act of the United Kingdom and the guidelines of the National Institutes of Health of the United States.

### Statistical analysis

GraphPad Prism (v8.0, GraphPad Software, USA) and SPSS 25.0 were used to conduct all statistical analyses in this research (IBM, SPSS statistical software, USA). We used the chi-square and Fisher exact tests to examine the association between clinicopathological characteristics and PKM2 expression. Survival factors were assessed using multivariate COX regression analysis. OS was analyzed using a Kaplan-Meier and a log-rank test. To evaluate the differences between the two groups, we employed the Student t-test, while the one-way analysis of variance was used for multiple comparisons. All data were expressed as mean ± SD and were the result of no less than 3 independent experiments. The threshold for statistical significance was set at *P* < 0.05. *P < 0.05, **P < 0.01, ***P < 0.001, ****P < 0.0001, ns: there is no significance in statistics.

## Results

### The clinical value of PKM2 expression in LNMs and prognosis of HPC

**Fig. 1:**
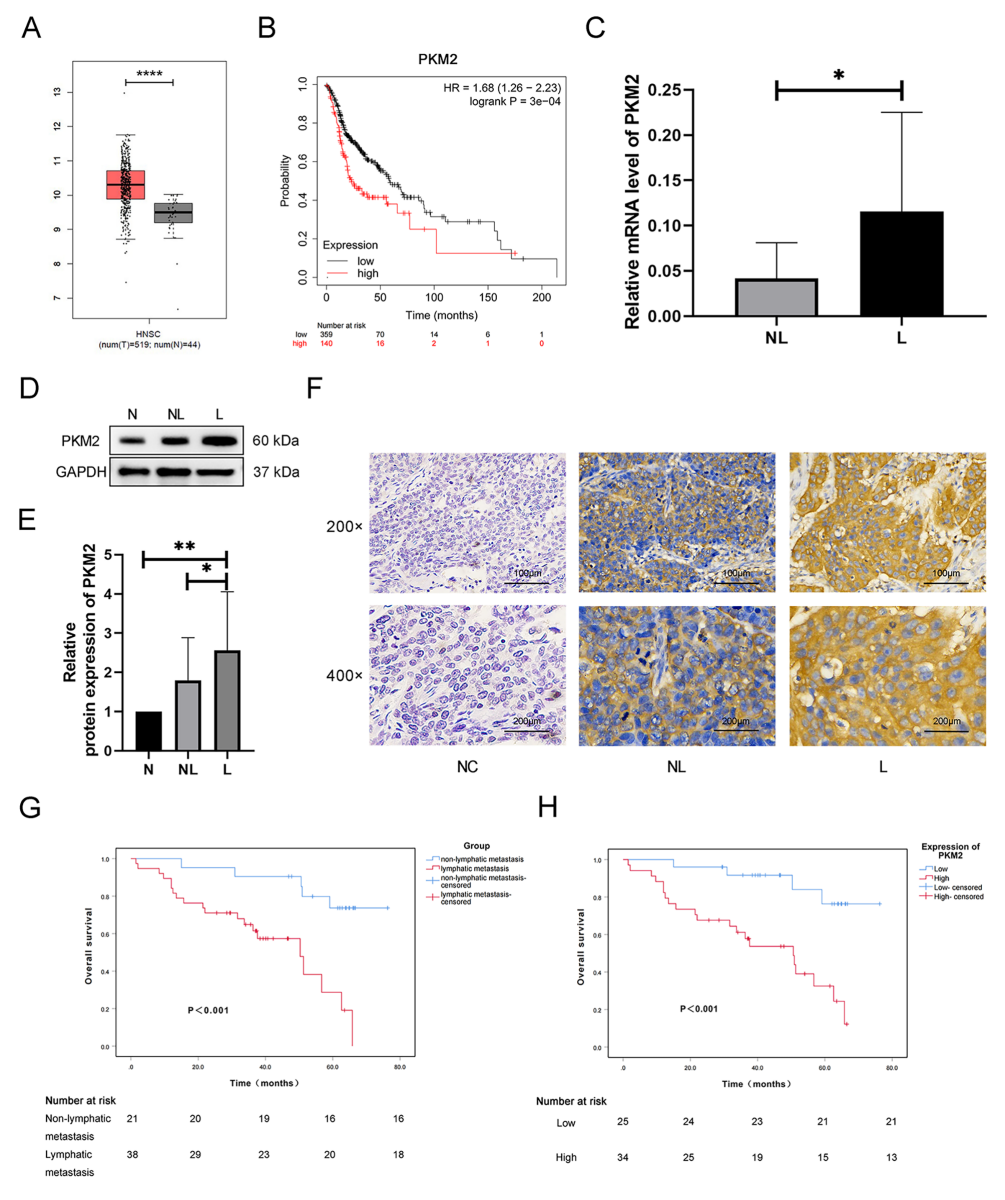
Expression and prognostic value analysis of PKM2 in clinical specimens. (**A**) Box plots show the PKM2 expression levels in 519 HNSCCs and 44 normal tissues by GEPIA2 online website. (**B**) The OS probability of HNSCC patients with high vs. low expression of PKM2 according to TCGA data. (**C**) qRT-PCR analysis of the expression of PKM2. (**D,E**) Western blotting analysis of the expression of PKM2. (**F**) Immunohistochemical staining for PKM2. (**G**) Kaplan-meier survival curve of of risk factor for lymphatic metastasis. (**H**) Kaplan-meier survival curve of of risk factor for PKM2 expression. L, lymphatic metastasis patients’ tumor tissue; NL, non-lymphatic metastasis patients’ tumor tissue; N, Normal tissue adjacent to the carcinoma; NC, negative control. *P < 0.05, **P < 0.01, ***P < 0.001

Our group has screened 2341 differentially expressed genes of HPC patients impacting LM by performing RNA sequencing on the original tumors of 5 patients with LM and 5 without LM. The gene *PKM2* is thought to be up-regulated in the LM subtype. To further understand PKM2’s function in HNSCC, we investigated its expression profile in 44 normal and 519 HNSCC tissues from the TCGA database. PKM2 expression is dramatically elevated in HNSCC tissues, as seen in Fig. 1A. This finding was further corroborated by Kaplan-Meier survival analysis, which found that increased PKM2 expression was related to a decreased likelihood of surviving in HNSCC patients (Fig. 1B). However, PKM2’s function in HPC remains unclear. HPC patients were examined in this research, and mRNA and protein levels of PKM2 were shown to be expressed in this disease. PKM2 mRNA levels were substantially greater in 14 patients with LM than in 14 patients without LM (*p* < 0.05) (Fig. 1C). When compared to the two other groups (5 patients without LM and 5 patients with similar nearby normal tissue), the PKM2 protein level in the group with LM was significantly greater (*p* < 0.05) (Fig. 1D, E).

IHC was used to identify PKM2 expression in 59 individuals with HPC. PKM2 staining was shown to vary across tissues of HPC, with stronger staining in LM than in non-lymphatic ones (Fig. 1F). PKM2 was expressed more often in the group that had LM (26/38, 68.4%) than in the group that did not (8/21, 38.1%) (Fig. 1F; Table 3). In this study, we looked at 59 patients with HPC to see if PKM2 expression was linked to any clinical features. We found that PKM2 expression was significantly related to LM (*p* < 0.05) but not with other factors like age, gender, smoking status, pathological stage, or pathological differentiation (Table 3). High PKM2 expression and LM were shown to be independent RFs for the prognosis of HPC using the Kaplan-Meier, log-rank test, and COX analysis (Fig. 1G, H; Table 4). These findings indicate that PKM2 expression level is significantly related to LM and poor prognosis.

**Table 3:** Association between PKM2 expression and clinicopathological characteristics of patients with hypopharyngeal carcinoma

Features	Variables	Expression of PKM2		*P* value
		High	Low	
Age				0.498
	≥ 60	22	14	
	< 60	12	11	
Smoking				0.749
	Yes	32	23	
	No	2	2	
Gender				1.000
	Male	33	25	
	Female	1	0	
Pathological stage				0.113
	Early (T1-T2)	5	8	
	Advanced (T3-T4)	29	17	
Lymphatic metastasis				0.024*
	Presence	26	12	
	Absence	8	13	
Pathological differentiation				0.343
	Low	6	7	
	Moderate and high	28	18	

*P < 0.05

**Table 4:** Univariate and multivariate Cox regression analyses of overall survival in hypopharyngeal carcinoma patients

Variable	Univariate analyses			Multivariate analyses		
	HR	95%CI	*P* value	HR	95%CI	*P* value
Age	1.270	0.545–2.959	0.579			
Smoking	23.005	0.033-15986.427	0.348			
Gender	21.037	0.000-3708102.146	0.621			
Pathological stage	1.790	0.532–6.030	0.347			
Lymphatic metastasis	5.650	2.007–15.907	0.001**	3.355	1.175–9.584	0.024*
Pathological differentiation	0.607	0.261–1.411	0.246			
Expression of PKM2	5.908	2.010-17.368	0.001**	3.935	1.281–12.090	0.017*

*P < 0.05, **P < 0.01

### PKM2 promoted the proliferation, migration, and invasion and inhibited the apoptosis of hypopharyngeal carcinoma cells in vitro

**Fig. 2:**
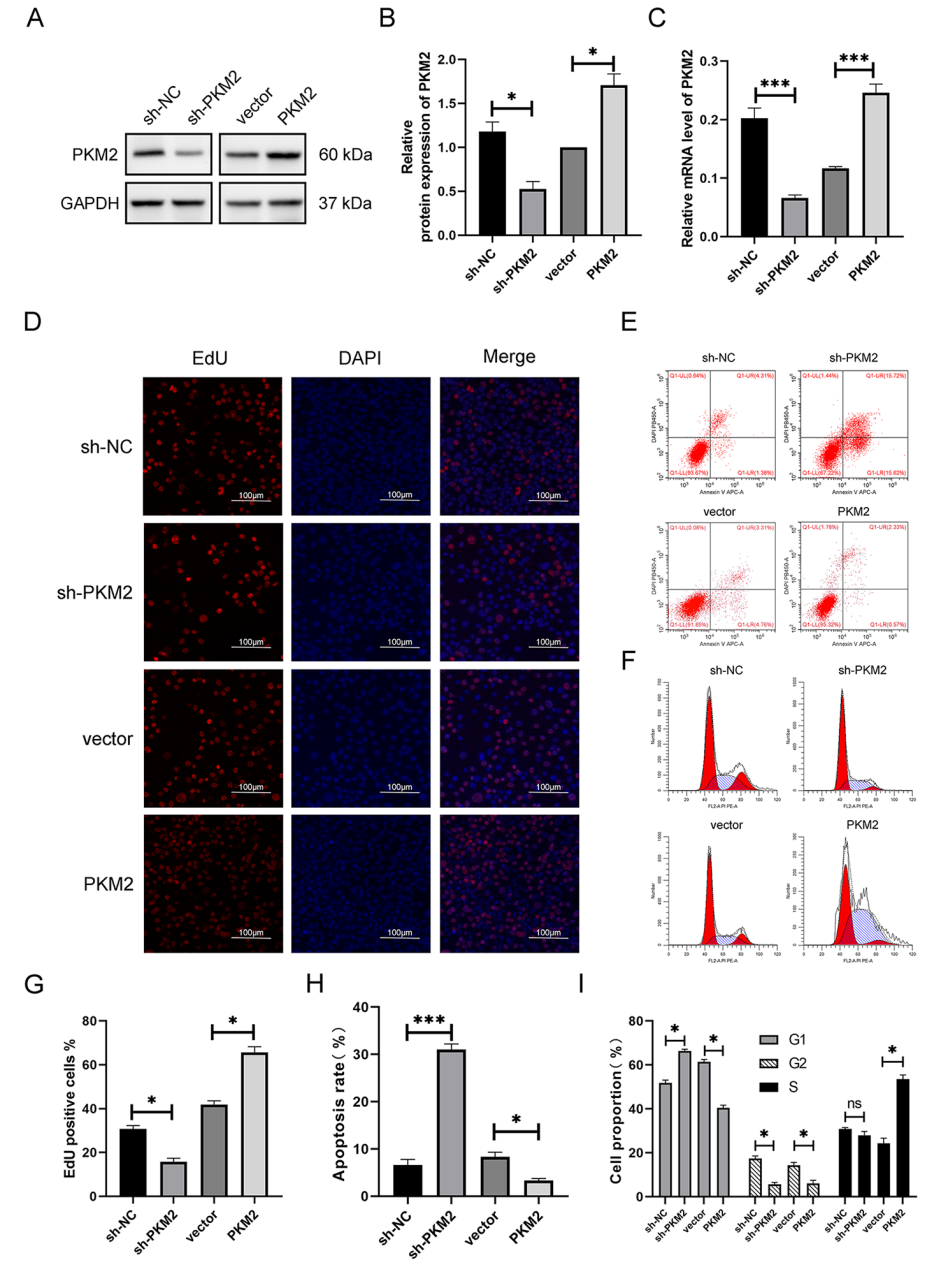
Expression of PKM2 in cell lines, cell proliferation assay and flow cytometry assay. (**A**, **B**) Western blotting analysis of the expression of PKM2 in FaDu cell line. (**C**) qRT-PCR analysis of the expression of PKM2 in FaDu cell line. (**D**) EdU proliferation assay in FaDu cell line. (**E**) Apoptosis assay in FaDu cell lines. (**F**) Cell cycle assay in FaDu cell lines. (**G**) Results analysis of EdU assay. (**H**) Results analysis of Apoptosis assay. (**I**) Results analysis of Cell cycle assay. *P < 0.05, **P < 0.01, ***P < 0.001

PKM2 was knocked down and overexpressed in FaDu cell lines using lentivirus shRNA and overexpression vector transfection. The PKM2 expression levels were measured by western blotting and qRT-PCR (Fig. 2A-C) and found to be considerably lower in the knockdown group (sh-PKM2) and higher in the overexpression group (PKM2) as compared to the negative control group (sh-NC and vector). The function of PKM2 in FaDu cell proliferation was determined using an EdU test. The findings revealed that decreasing PKM2 expression decreased cell proliferation (Fig. 2D, G), whereas overexpressing PKM2 increased cell proliferation. As measured by FCM, the apoptosis rate was considerably lower in the PKM2 and higher in sh-PKM2 than in the sh-NC (Fig. 2E, H). In sh-PKM2, the proportion of cells in the G1 phase increased while in the G2 phase dropped. However, the PKM2 demonstrated the inverse pattern; the proportion of cells in the S phase rose while in G1 declined (Fig. 2F, I). In conclusion, it has been shown that PKM2 expression may facilitate tumor development by increasing cell proliferation, inhibiting apoptosis, and regulating the cell cycle in tumor cells.

**Fig. 3:**
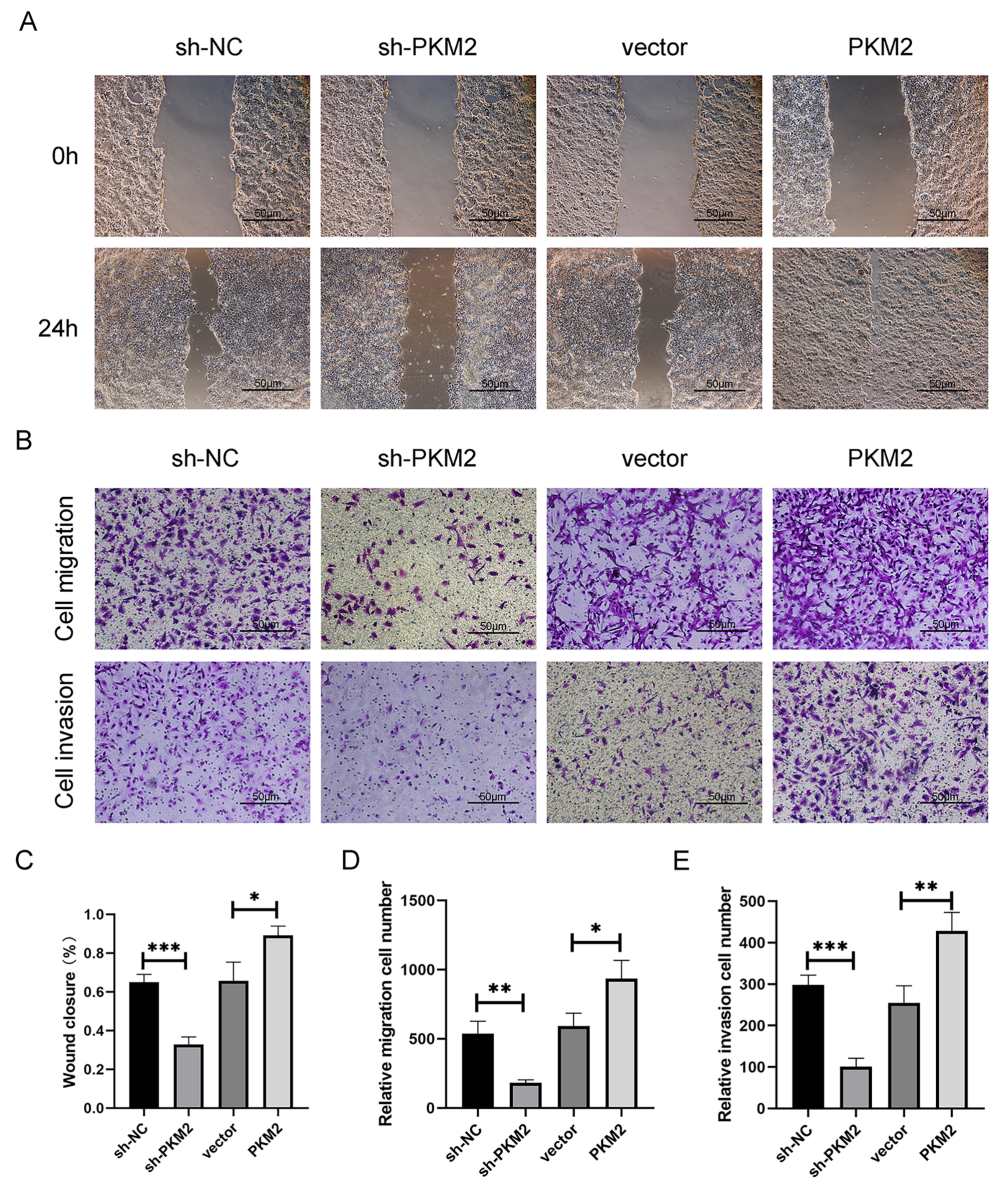
Wound Healing assay and Transwell assay. (**A**) Wound Healing assay in FaDu cell line. (**B**) Cell migration and invasion assay in FaDu cell line. (**C**) Results analysis of Wound Healing assay. (**D,E**) Results analysis of Transwell assay. *P < 0.05, **P < 0.01, ***P < 0.001

LM is a multistep process involving many alterations, such as cell migration and enhanced invasive potential. Metastasis from HPC is common in clinical practice. We employed the Transwell and wound healing assay to test whether PKM2 influenced FaDu cells’ migration and invasion. Overexpression of PKM2 promoted FaDu cell migration, whereas down-regulation of PKM2 reduced FaDu cell migration in wound-healing assays (Fig. 3A,C). The Transwell experiment corroborated these findings, showing that FaDu cell migration and invasion were greatly decreased when PKM2 expression was down-regulated. However, this result was contrary to that of FaDu cells in the overexpressed group (Fig. 3B,D,E). When considered together, these findings raise the intriguing possibility that PKM2 promotes the migration and aggressiveness of HPC cells.

### Down-regulating PKM2 can inhibit tumor growth and lymphatic metastasis in vivo

**Fig. 4:**
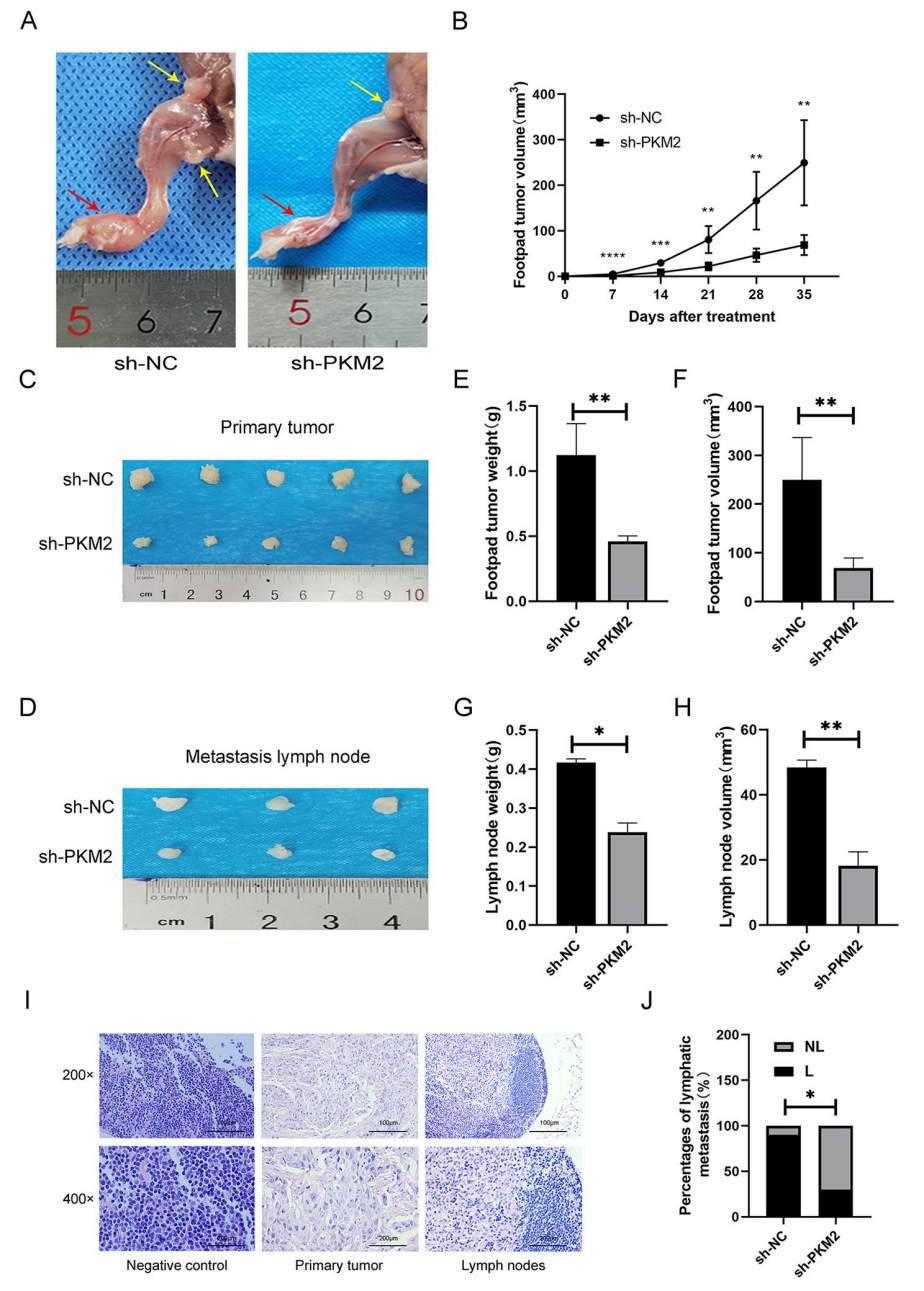
PKM2 regulates tumor growth and lymphatic metastasis in vivo. (**A**) Anatomy of foot-pad primary tumors and metastatic LNs in nude mice, red arrow indicates foot-pad primary tumors, yellow arrow indicates metastatic LNs. (**B**) Tumor growth graphs of nude mice. (**C**) Representative images of enucleated footpad tumors for the indicated groups. (**D**) Representative images of enucleated popliteal LNs for the indicated groups. (**E,F**) The weight and volume of footpad tumors for the relevant groups. (**G,H**) The weight and volume of LNs for the relevant groups. (**I**) Typical H&E-stained micrographs of primary tumors and metastatic LNs in vivo. (**J**) Percentages of lymphatic metastasis in animal models. *P < 0.05, **P < 0.01, ***P < 0.001

Nude mice were used to create a footpad tumor inguinal popliteal lymphatic metastasis model (Fig. 4A). To study the influence of PKM2 level on the development and LM of HPC in vivo, 10 male BALB/c mice were inoculated with either sh-PKM2 or sh-NC cells in the footpads. It were fed in a specific, pathogen-free environment for five weeks. After the mice were anesthetized and killed, the primary pad tumors and metastatic lymph nodes in the inguinal popliteal fossa were collected (Fig. 4C-D). At the same time, tumor weight and volume were measured. In comparison with the sh-NC group, the tumors in the sh-PKM2 group grew at a slower rate (Fig. 4B), and the pad tumors and inguinal popliteal lymph nodes in the sh-PKM2 group were both lighter and smaller (Fig. 4E-H). Metastatic lymph nodes were counted in the groin and popliteal fossa, and the primary tumor in the foot pad was confirmed by HE staining (Fig. 4I). 30% of mice in the sh-PKM2 group and 90% of animals in the sh-NC group had LM (Fig. 4J). The rate at which cancer spread to lymph nodes was reduced in the sh-PKM2 group than in the sh-NC group. It is suggested that down-regulated PKM2 inhibits tumorigenesis and LM of HPC in vivo.

### PKM2 promotes lymphatic metastasis through EMT induction

**Fig. 5:**
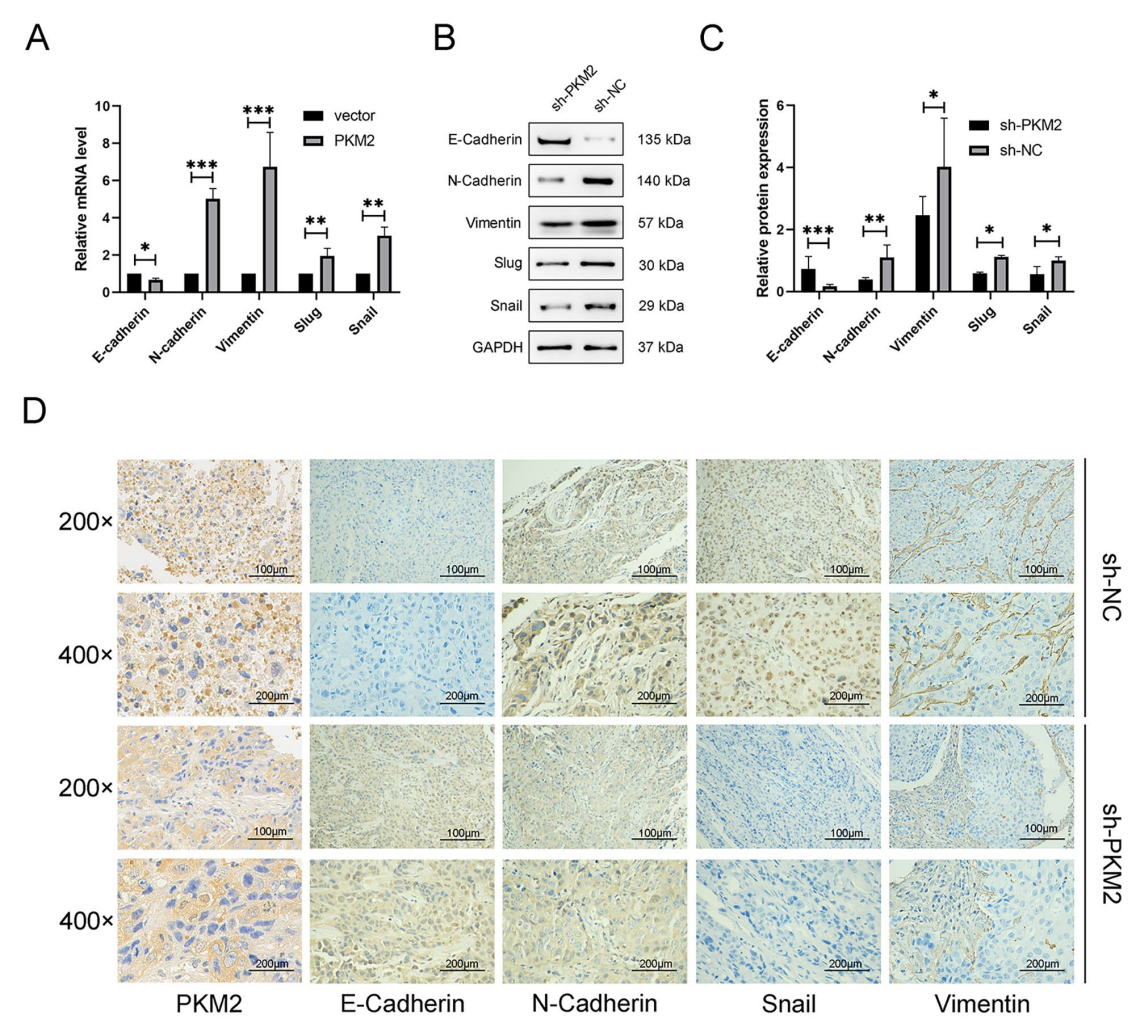
PKM2 promotes lymphatic metastasis through EMT induction. (**A**) qRT-PCR analysis of mRNA levels of key EMT-related genes in vector and PKM2 groups in vitro. (**B,C**) Western blotting analysis of protein levels of EMT marker proteins in footpad tumors sh-PKM2 and sh-NC groups in vivo. (**D**) Typical micrographs of IHC-stained in footpad tumors sh-PKM2 and sh-NC groups in vivo. *P < 0.05, **P < 0.01, ***P < 0.001

In recent years, our team has conducted a series of studies on LM of HPC, and these studies have shown that EMT is a critical step in the initiation of tumor metastasis [17, 19]. To further investigate the mechanism behind the lymphatic metastasis mediated by PKM2, using qRT-PCR to uncover the mRNA level of EMT-related genes in the FaDu cell lines of vector and PKM2, the E-cadherin level in the PKM2 group was lower, and that of N-cadherin, Vimentin, Slug, Snail was higher (Fig. 5A). At the same time, expression of E-cadherin, N-cadherin, and other EMT markers in malignancies of the footpad was examined using western blotting and IHC. Tumors in the sh-PKM2 group were shown to have lower levels of PKM2, N-cadherin, Vimentin, Snail, and others, and greater levels of E-cadherin compared to those in the sh-NC group (Fig. 5B-D). Therefore, based on in vitro and in vivo monitoring, we speculate that PKM2 promotes LM of HPC through EMT induction.

## Discussion

Approximately 0.8-1.5% of all head and neck cancers are HPC, an extremely aggressive squamous cell carcinoma [30]. Due to its unique anatomical structure, HPC is notoriously difficult to detect in its early stages, prolonging the treatment process and increasing the likelihood of the disease spreading to lymph nodes. Studies have shown that 60-80% of patients with HPC will develop metastases in their cervical lymph nodes, while as much as 30% of patients at the cN0 stage would have occult LNMs [31]. Diameter, number, and extra invasion of metastatic lymph nodes are all effect variables for the prognosis of HPC, as has been proven in previous research [32, 33]. In addition, the 5-year OS rates for patients with LM with either extra invasion or without extra invasion are 29.9% and 62.5%, respectively [34]. As a result, improving the therapeutic impact of HPC necessitates a greater understanding of the role LM plays in prognosis and the development of appropriate treatment strategies.

To further understand PKM2’s involvement in HNSCC, we compared its expression in 44 normal and 519 HNSCC tissues from the TCGA database and found that PKM2 was considerably overexpressed in HNSCC. Afterward, RT-PCR was employed to examine the detectable expression variations. The findings revealed that PKM2 expression was significantly greater in LM than in non-LM patients. Western blotting and immunohistochemistry also confirmed the expression of PKM2. Overall, the experimental findings agreed with the RT-PCR analysis. The Kaplan-Meier survival analysis also disclosed that patients with elevated PKM2 expression had a lower 5-year OS rate. Kaplan-Meier, log-rank, and COX tests all agreed that PKM2 expression is an independent RF influencing the HPC prognosis. This data supports the hypothesis that PKM2 is a potential biomarker for the early diagnosis, prevention, and treatment of LM in patients with HPC.

PKM2 is an essential glycolytic enzyme that is often up-regulated in tumors. Cancer development and metastasis rely heavily on glycolysis for their energy needs. When mitochondrial oxidative phosphorylation gives way to aerobic glycolysis, this metabolic shift is called the Warburg effect. To satisfy the metabolic requirements for tumor growth, PKM2 plays a crucial part in the Warburg effect, a cancer-specific glycolytic pathway that provides tumor cells with energy rapidly to proliferate, migrate, and invade. PKM2 has been linked to cancer, particularly HPC; however, its specific carcinogenic activities are unknown. Here, we used the lentivirus-mediated knockdown and overexpression approaches to ascertain the biological functions of PKM2 in FaDu cell lines. As we hypothesized, our in vitro findings showed that PKM2 is pivotal in proliferation and apoptosis. Given the established involvement of PKM2 in supporting neoplastic development in malignancies by supplying metabolic intermediates, our results are not too unexpected. We also discovered that PKM2 knockdown significantly reduced cell invasion and migration. Increased cell invasion and migration were among the several effects of overexpressing PKM2. Our results propose that PKM2 aids in the development of HPC by regulating cell proliferation, apoptosis, and migration. An aggressive clinicopathological profile and poor patient prognosis are linked to its overexpression in a subset of HPC cases. The intriguing natural chemical β-elemene suppresses breast cancer metastasis by inhibiting dimeric PKM2 transformation and nuclear translocation-mediated aerobic glycolysis [35]. The use of PKM2 inhibitors, either naturally occurring or synthetically produced, has shown promise as a method for halting the metastasis and proliferation of cancer. Cancer treatment using PKM2 inhibitors shows great promise [36].

Cancer often exhibits its malignant nature by promoting invasion and metastasis. For all malignancies, metastasis is the ultimate form of cancer. Metastases from primary tumors to distant normal tissues account for more than 90% of the death rate associated with solid tumors [37]. Metastasis from carcinoma is tightly linked to EMT, making it a key cause of cancer development [27]. Snail, Slug, ZEB1, and Twist are only a few EMT-TFs that may suppress E-cadherin expression and increase cancer cell motility and invasiveness [38]. The EMT-TFs that promote a protumorigenic environment by up-regulating the production of mesenchymal proteins are directly responsible for the tumor’s ability to invade and metastasize [39, 40]. Activation and expression of PKM2 in various malignancies promotes EMT and, thus, tumor metastasis [24, 25].

Our research included the FaDu cell lines transfection using lentivirus shRNA and an overexpression vector to reduce PKM2 expression and increase its levels, respectively. Stable PKM2 knockdown in FaDu cells suppressed tumor cell proliferation, migration, and invasion, while increased PKM2 levels in FaDu cells had the opposite effect. Overexpression of PKM2 markedly enhanced expression of E-cadherin and inhibited N-cadherin, Slug, and Snail expression, while its silencing had the opposing effects, suggesting that PKM2 promotes the invasion, migration, and proliferation capacity of these cells through induction of EMT. These findings show that PKM2 enhances EMT/cell motility and, consequently, cancer metastasis and its critical involvement in cell proliferation. Limitations of the current study include a small sample size and an insufficient investigation of the underlying molecular mechanism, which will be addressed in future research.

## Conclusions

In conclusion, our results show that PKM2 enhances EMT and cell metastasis and provide evidence for its clinical and biological role in aiding LM in HPC. Based on these findings, PKM2 has promise as a predictive biomarker of LNMs and a therapeutic target for people with HPC. The PKM2-targeting drugs that are both highly selective and relatively non-toxic provide promising therapeutic options for cancer treatment.

## Data Availability

The original contributions presented in the study are included in the article/supplementary material, further inquiries can be directed to the corresponding author.

## Abbreviations

HPC: Hypopharyngeal carcinoma
HNSCC: Head and neck squamous cell carcinoma
LM: Lymphatic metastasis
LN: Lymph node
LNMs: Lymph node metastasis
PKM2: Pyruvate kinase M2
OS: Overall survival
TCGA: The Cancer Genome Atlas
qRT-PCR: Quantitative real-time-polymerase chain reaction
GAPDH: Glyceraldehyde 3-phosphate dehydrogenase
IHC: Immunohistochemical
RF: Risk factor
FBS: Fetal bovine serum
PBS: Phosphate-buffered saline
sh-RNA: Short hairpin RNA
NC: Negative control
EMT: Epithelial Mesenchymal Transition
